# Dementia awareness raising forum: Improving attitudes towards people living with dementia

**DOI:** 10.1177/14713012241272852

**Published:** 2024-08-25

**Authors:** Sheridan T. Read, Rosemary Saunders, Matthew A. Albrecht, Ravani Duggan

**Affiliations:** School of Nursing and Midwifery, 2498 Edith Cowan University, Australia; School of Nursing and Midwifery, 2498 Edith Cowan University, Australia; School of Psychological Science, Western Australia Centre for Road Safety Research, University of Western Australia, Australia; School of Nursing, Midwifery and Paramedicine, 1649 Curtin University, Australia

**Keywords:** dementia, stigma, attitudes, communities, education forum

## Abstract

Stigma surrounding dementia is a significant issue affecting individuals and communities leading to discrimination towards those living with the condition. However, the changing paradigm in dementia support to living well with dementia can reduce this stigma and improve community attitudes. A community initiative aimed to address this evaluated the impact of a two-hour education forum involving 92 community members. Presentations from experts, including a person with dementia, addressed dementia-related human rights issues and initiatives to live well with dementia. Attendees completed the new Dementia Community Attitudes Questionnaire (DCAQ) aligned with the evolving paradigm of living well with dementia before and after the forum. Participants with prior dementia education had higher initial scores while those without education showed more significant improvements. Almost all DCAQ items showed post-forum score improvements. This community Dementia Awareness Raising Forum provided an opportunity for people to come together and initiate conversations around dementia resulting in more positive community attitudes.

## Introduction and background

Historically, the experience of dementia has primarily been characterised as one of loss (Edvardsson et al., 2008) but the focus is changing to living well with dementia and respecting the decision-making autonomy of the individual (de Boer et al., 2012; Dening et al., 2017; Karel et al., 2007). The World Health Organisation (WHO) promotes a human rights-based approach for people with dementia to safeguard their right to experience an adequate standard of living, social inclusion and rehabilitation opportunities to preserve their autonomy for as long as possible (World Health Organisation, 2017; World Health Organization, 2015). People with dementia have a right to all opportunities afforded to people including their right to respect and justice (Cahill, 2020). A rights-based approach is one that ensures the person is valued, their uniqueness considered, and service provision is person-centred and needs-based (Brooker, 2003, 2007; Kitwood, 1993, 1997). Ensuring the human rights of this cohort of people are upheld is integral to their sustained wellbeing.

Opportunities for people with dementia to live well are often limited due to dementia-related stigma that results in people diagnosed frequently being devalued and denied opportunity to live well due to the widespread belief that little can be done to support them (Gove et al., 2016). Such stigmatising attitudes mean people with dementia may have poor help-seeking behaviours resulting in impaired coping ability (Vernooij-Dassen et al., 2005). Failure to seek help impacts timely diagnosis and delays commencement of treatment interventions related to lifestyle behaviour changes that can be implemented to delay cognitive decline, and other strategies to live well (Prince et al., 2011). Living well with dementia is a relatively new phenomenon that needs further promotion to help overcome related stigma and enhance the wellbeing of those diagnosed (Quinn et al., 2022). People with dementia report living well as sustaining connection to their pre-diagnosis lifestyle such as joining community social groups or acquiring less demanding work roles (Read et al., 2016).

The WHO’s public health response to dementia promotes the need for communities that are dementia-friendly to ensure the human rights and wellbeing of people with dementia are upheld (World Health Organisation, 2017). A dementia-friendly community is a community that holds positive attitudes towards people diagnosed (Green & Lakey, 2013). Dementia inclusive community-based solutions that reduce stigma and empower people with the condition to remain independent and emphasise their potential are needed. Such solutions include the creation of easy to navigate environments, awareness raising around responding to the needs of people with dementia within the community and offering people with dementia reablement opportunities (Green & Lakey, 2013). A reablement approach is goals oriented and maximises a person with dementia’s independence central to their wellbeing (Jeon et al., 2018, 2019; Poulos et al., 2017).

Awareness raising initiatives and research are required to broaden community understanding of what it means to live with dementia in the 21st century and promote positive attitudes towards people with the condition (Phillipson et al., 2019). Evaluating the effectiveness of such initiatives, and disseminating research findings are some of the mechanisms to achieve this (Cowan, 2021). Attitudes to dementia are reflected in what people know about the condition, how they feel about people living with dementia and how they respond when speaking with or about people diagnosed. Assisting people to integrate their new and pre-existing knowledge provides them an opportunity to review how they feel about and respond to this cohort of people (Olson & Kendrick, 2012). Therefore, evaluating awareness-raising initiatives, such as the Dementia Awareness Raising Forum reported in this paper and conducted as part of a larger study, or Community Conversation forums is essential to establish what further information people need to facilitate dementia related attitudinal change. A Community Conversation forum brings together members of the community, utilising expertise to address issues of significance, is often facilitated and can be a means of investigation (Trainor, 2018). The aim of this study was to evaluate the impact of a Dementia Awareness Raising Forum designed to promote more positive attitudes to dementia.

## Methodology

### Intervention

A facilitated, two-hour Dementia Awareness Raising Forum, titled, Dementia: Living Well and Staying Connected was held with community members in a metropolitan area in Western Australia in 2019 in partnership with the Curtin Ageing Research Network (a multidisciplinary collaboration of researchers from Curtin University) and Alzheimer’s Western Australia. The structure of the forum encouraged a supportive environment that included an introduction describing the program and an invitation for participants to ask questions in a final Q and A session. Food and beverage were served prior to the commencement of proceedings. Information was presented at the forum by experts in the field, including a person living with dementia, detailing the consumer voice-on the experiences and expectations of people living with the condition (Read et al., 2016). The importance of considering the dementia human rights movement was conveyed to attendees, in particular, specifying the Convention on the Rights of Persons with Disabilities (United Nations, 2006). Information on new knowledge regarding early diagnosis was provided, plus new initiatives in living well with dementia promoting a wellness approach (Power, 2014, 2017). This enabled attendees to develop an awareness and understanding of what it means to be living with a dementia diagnosis and how a person diagnosed can be supported to maintain their quality of life. Participants were members of the community who had responded to an email invitation to attend and included people on the mailing list of related community organisations such as Alzheimer’s and Carers WA, past and present Curtin University staff and students, and employees of aged care community organisations, public and private hospitals.

### Evaluation

The evaluation aimed to measure the impact of the Dementia Awareness Raising Forum addressing attitudes towards people living with dementia through the implementation of the contemporary Dementia Community Attitudes Questionnaire (DCAQ). The DCAQ is a ten-item tool, with a ten-point Likert type scale, newly developed to align with the current context of living with dementia, respecting autonomy and community participation of people diagnosed despite challenges they need to overcome (Read et al., 2020). Demographic details including dementia-related education, either formal through tertiary education, or informal through community support organisations; or any dementia related experience, either as a person living with dementia or alternatively knowing or supporting a person with dementia is a part of the tool and were collected from participating attendees. No identifying information was requested, and participants remained anonymous. The questionnaire collected both pre and post forum data and participants were instructed to complete the pre-forum questions before the forum started and the post-forum questions at the end of the forum.

### Ethics approval

Ethics approval was obtained from the Curtin University Human Research Ethics Committee, HR139/2013. Participants responded to an email invite and voluntarily attended the forum. For attendees who elected to participate in the forum evaluation, completion of the questionnaire was voluntary and consent to participate was implied through completing and returning the questionnaire. Details of a counselling service were provided to attendees as it was noted that the forum content had the potential to cause discomfort for participants with a personal experience of dementia.

### Data analysis

Data were entered into the IBM Statistical Package for Social Sciences (SPSS) version 25 for analysis to determine differences in item scores between participants with and without prior dementia education pre- and post-forum. Descriptive statistics summarised characteristics of attendees. An overall score was calculated by averaging participants’ responses to all items of the questionnaire (including reverse scoring where appropriate) and this was analysed before assessing individual items. Independent samples T Tests were used to contrast baseline differences between participants with versus without dementia-related education. Two-way repeated-measures analysis of variance (ANOVA) were used to determine the overall effect of the intervention (intervention effect: pre-vs. post-forum scores [within-subjects factor]), and whether there was an interaction of the intervention with education (comparing participants with vs. without dementia education [between-subjects factor]). Missing data for the 10 questionnaire items comprised less than 1% of data for both the pre-test and post-test responses and were imputed using the standard imputation procedure in SPSS, which uses a regression-based approach. There was one participant with a missing value for the dementia-related education variable; this value was not imputed, the value was omitted from the analyses comparing those with pre-existing dementia-related education to those without. Alpha was set at .05.

## Results

### Demographic background of participants

Questionnaire responses were received from 92 out of 112 attendees, an 82% response rate. Participants’ ages ranged from 22 to 82 years (*M* = 51.01, *SD* = 14), there were 17 (18.5%) males and 75 (81.5%) females. One person (1.1%) indicated they had dementia, 14 (15.2%) indicated no dementia-related experience, 19 (20.7%) knew someone with dementia, and 58 (63%) had a family member/friend experiencing dementia and/or supported a person with dementia (home/work). While 33 (35.9%) attendees indicated they had no dementia-related education, 48 (63%) reported formal work-related education, and 10 (10.8%) had received informal education to help them support family/friends with dementia.

### Baseline attitudes of participants

At baseline participants with dementia education scored significantly better on the overall scale score (t_89_ = 6.6, *p* < .001) and on all items except for Item 10 (which related to a dementia diagnosis being useful in informing planning for the future), see Table 1. There were no differences at baseline between male and female participants.

**Table 1.** Baseline questionnaire differences by education.

	Participants with prior dementia-related education (*n* = 58)		Participants with no dementia-related education (*n* = 33)						
Question	**Mean**	**Standard Deviation**	**Mean**	**Standard Deviation**	**Mean Diff**	**SD Diff**	**T – Value**	***p*-value**	**Confidence Interval**
**Q1. I Have a good understanding of what it would be like to live with dementia**	6.73	2.14	4.64	1.93	2.05	.44	4.65	<0.001	1.17, 2.93
Q2. * The main symptom of dementia is always memory loss	5.69	2.74	4.45	1.89	1.24	0.49	2.53	.013	0.27, 2.20
Q3. * Medication is the only treatment that can reduce symptoms related to dementia	7.83	1.57	6.23	1.94	1.62	0.39	4.15	<0.001	0.84, 2.40
**Q4. There is a range of strategies that can help people with dementia in their everyday life**	9.22	1.01	7.74	1.85	1.45	0.34	4.21	<0.001	0.76, 2.15
**Q5. I Have the potential to improve the lives of people living with dementia**	8.83	1.35	6.67	2.05	2.12	0.39	5.38	<0.001	1.33, 2.91
**Q6. People with dementia can contribute substantially to their community**	8.24	1.66	6.52	1.87	1.75	0.39	4.53	<0.001	0.98, 2.53
**Q7. Maintaining independence is one way to help a person living with dementia**	8.53	1.55	7.15	2.12	1.38	0.42	3.30	<0.002	0.54, 2.23
Q8. * People with dementia need assistance all of the time	6.88	1.87	5.72	1.71	1.17	0.40	2.96	<0.004	0.38, 1.96
**Q9. People with dementia have the right to be involved in supported decision making about their future**	9.31	1.19	7.85	1.94	1.46	0.37	3.93	<0.001	0.71, 2.21
**Q10. For people with symptoms of dementia, a diagnosis can inform planning for the future**	8.74	1.74	8.61	1.34	0.14	0.33	0.41	0.68	−0.52, 0.83

*Note.* * denotes that the statement is designated incorrect and has been reversed scored.

### Impact of the dementia awareness raising forum

As an outcome of the Dementia Awareness Raising Forum, there was an increase in the overall score from pre - to post forum (F_1, 89_ = 58.0, *p* < .001, partial-η^2^ = 0.39) and an interaction between time and education (F_1,89_ = 17.1, *p* < .001, partial-η^2^ = 0.16). Items 2, 4, 5, 6, 7 and 9 (see Table 2) demonstrated a statistically significant interaction between time (pre vs. post) and education (with Items 7 and 9 surviving corrections for multiple comparisons using the Holm-Bonferroni method), indicating that forum attendees with no prior dementia education showed a greater improvement in post-forum scores compared to attendees with dementia education on these items. Items 1, 3, and 10 (see Table 2) did however, show significant overall effects of the intervention (items 1 and 10 surviving corrections for multiple comparisons), with scores increasing following the forum. Only Item 8 did not show any significant improvement in main effect or interaction effect, Item 4 showed significant improvement in interaction effect only.

**Table 2.** Intervention and Interaction effect.

	Time (Pre vs. Post) – main effect of intervention					Time by education – interaction effect				
	df_n_	df_d_	F Value	P value (+Holm-corrected)	Partial-η^2^	df_n_	df_d_	F Value	P value (+Holm-corrected)	Partial-η^2^
Q 1 I have a good understanding of what it would be like to live with dementia	1	89	33.3	<0.001 (<0.001)	0.27	1	89	3.4	0.068 (0.27)	0.037
Q 2 * The main symptom of dementia is always memory loss	1	89	16.0	<0.001 (0.003)	0.15	1	89	4.2	0.043 (0.25)	0.045
Q 3 * Medication is the only treatment that can reduce symptoms related to dementia	1	89	7.1	0.009 (0.070)	0.074	1	89	2.7	0.11 (0.32)	0.029
Q 4 There is a range of strategies that can help people with dementia in their everyday life	1	89	3.0	0.087 (0.26)	0.033	1	89	4.6	0.035 (0.25)	0.049
Q 5 I have the potential to improve the lives of people living with dementia	1	89	10.9	0.001 (0.033)	0.11	1	89	6.6	0.012 (0.10)	0.069
Q 6 People with dementia can contribute substantially to their community	1	89	40.0	<0.001 (<0.001)	0.31	1	89	4.6	0.035 (0.25)	0.049
Q 7 Maintaining independence is one way to help a person living with dementia	1	89	47.2	<0.001 (<0.001)	0.35	1	89	10.4	0.002 (0.016)	0.11
Q 8 * People with dementia need assistance all of the time	1	89	2.5	0.12 (0.26)	0.027	1	89	0.12	0.72 (1.00)	0.001
Q 9 People with dementia have the right to be involved in supported decision making about their future	1	89	21.1	<0.001 (0.002)	0.19	1	89	14.9	<0.001 (0.002)	0.14
Q 10 For people with symptoms of dementia, a diagnosis can inform planning for the future	1	89	15.0	<0.001 (0.002)	0.14	1	89	0.36	0.55 (1.00)	0.004

*Note.* * denotes that the statement is designated incorrect and has been reversed scored.

These findings prove the effectiveness of the Dementia Awareness Raising Forum which resulted in more positive community attitudes towards dementia overall. Whilst previous dementia-related education positively impacted baseline scores, a significant change was seen in attendees with no prior dementia education, with the forum increasing knowledge and influencing attitudes.

## Discussion

The findings suggest that the Dementia Awareness Raising Forum engaged participants and resulted in an immediate positive change in community attitudes towards people with dementia. Only two items (Items 4 - There is a range of strategies that can help people with dementia in their everyday life and 8 - People with dementia need assistance all of the time), showed no statistically significant main effect of the forum. Item 4 did show a significant time by education interaction. The reason for the limited impact in these areas may relate to pre-existing knowledge levels (46.7% having previous formal or informal dementia-related education), given the relatively high number of correct pre-forum responses, or to limited coverage of the topic during the forum.

Content delivered at the forum was contemporary in nature designed to highlight to attendees the changing paradigm of how people living with dementia wish to be supported (Read et al., 2016). While it would be expected that attendees with dementia education score significantly better on all items on the pre-forum questionnaire (Table 1), this was an exception for Item 10 (For people with symptoms of dementia, a diagnosis can inform planning for the future). This is a significant finding as it indicates potential limitations within current dementia education programs that fail to inform on the expectations of people with dementia to remain central to decision making regarding their future (Read et al., 2016).

Participants with no dementia-related education (35.9% of respondents) had significantly larger improvements in their scores post-forum compared to those with dementia education for Items 2 (The main symptom of dementia is always memory loss), 4 (There is a range of strategies that can help people with dementia in their everyday life), 5 (I have the potential to improve the lives of people living with dementia), 6 (People with dementia can contribute substantially to their community), 7 (Maintaining independence is one way to help a person living with dementia) and 9 (People with dementia have the right to be involved in supported decision making about their future) (Table 2 - interaction effect). These items sought to measure people’s awareness about what it means to be living with dementia today, while also addressing the benefits of community engagement and independence. These topics were covered explicitly during speaker presentations, further enhanced by including a person living with dementia which demonstrated their ability to contribute despite their dementia diagnosis. Findings supported the expectations that attendees with dementia-related education would have a better grasp on these issues than the group without previous education and highlights the impact that education initiatives can have in creating more dementia-friendly communities. To enhance community awareness and attitudes towards people with dementia, information presented at forums needs to target participants depending on their level of dementia-related education. E-Learning web-based education modules that customise the information presented based on learner pre-existing knowledge, may prove to be an effective alternative (Eccleston et al., 2019; Peisah et al., 2016) to face-to-face offerings.

While results of this study had a positive outcome with improved community attitudes post forum it is unclear whether such initiatives are the most effective means by which to create attitude change. There needs be some consideration of alternative interventions which may prove more effective. For example, Goldman and Trommer (2019) report on an experiential learning intervention for medical students that resulted in attitude change following their extended contact with a person with dementia. Small face to face discussion groups with activity based learning has also been shown to be effective in achieving attitudinal change (Surr et al., 2017). The outcome of the present study has raised questions as to whether awareness raising forums sustain attitude change over time. The study reported in this paper looked at immediate change in attitudes, hence it is unclear whether these were sustained beyond the event. Effectiveness of community awareness raising initiatives with a multi focused approach (education sessions, workshops, community engagement projects), such as, the raising awareness about ageing initiative, conducted in Pingelly, Western Australia (Baldassar et al., 2021) and the dementia friendly community project based in Kiama, New South Wales (Phillipson et al., 2019) also need to be deliberated. While such initiatives may be time and resource intensive, they may more likely sustain attitudinal change over the longer term. Further research should include establishing test-retest reliability of the DCAQ by administering the questionnaire more than once over a period with the same individuals to determine whether forums like the one reported in this paper have a prolonged impact on community attitudes.

A limitation of this evaluation is that despite a robust development process, the new Dementia Community Attitudes Questionnaire requires further testing to confirm its psychometric properties (Read et al., 2020). While Items 4 (There is a range of strategies that can help people with dementia in their everyday life), and 8 (People with dementia need assistance all of the time) showed no significant change for main effect, scores for these items did show some improvement post-forum meaning that a greater sample size may have resulted in significant change. Recruitment through established dementia networks and care facilities might mean that people who attended already had an existing exposure and knowledge level or were motivated to learn and therefore may not have represented the wider community where attitudes of stigma pervade. The collection of pre-post data on two sides of the same form could have led to potential bias in the results of the study as participants could have referred to their pre-forum answers, and due to social desirability, looked to demonstrate an improvement in their post-forum scores. This consideration is particularly significant given the speakers included a person with dementia and that some attendees at the session were from known networks of the forum convenor. It is recommended that further research is needed to test the questionnaire and also determine if any changes in attitudes and knowledge were sustained over time.

## Conclusion

Ongoing knowledge dissemination within the community about living well with dementia is critical to ensure that the autonomy of people with this condition is respected, support is provided appropriately, and community inclusiveness is demonstrated. Awareness raising initiatives can impact attitudes and positive attitude changes occurred following implementation of the Dementia Awareness Raising Forum.

## References

[bibr1-14713012241272852] Baldassar L. Krzyzowski L. Stevens C. Lozeva S. (2021). Raising awareness about ageing in rural and remote communities: An evaluation of an anti-ageism campaign in Pingelly. https://pingellysomersetalliance.com.au/wp-content/uploads/2021/12/Pingelly-Ageism-31-Oct-final-to-view-1.pdf.

[bibr2-14713012241272852] Brooker D. (2003). What is person centred care in dementia? Reviews in Clinical Gerontology, 13(3), 215–222. DOI: 10.1017/s095925980400108x.

[bibr3-14713012241272852] Brooker D. (2007). Person-centred dementia care: Making services better. Jessica Kingsley Publishers. 10.7748/nop.19.5.22.s21 27726617

[bibr4-14713012241272852] Cahill S. (2020). New analytical tools and frameworks to understand dementia: What can a human rights lens offer? Ageing and Society, 42(7), 1489–1498. DOI: 10.1017/S0144686X20001506.

[bibr5-14713012241272852] Cowan T. L. (2021). College students' and community members' attitudes toward dementia: The impact of dementia friends sessions. Gerontology & Geriatrics Education, 42(1), 140–149. DOI: 10.1080/02701960.2019.1657859. 31426726

[bibr6-14713012241272852] de Boer M. E. Dröes R. Jonker C. Eefsting J. A. Hertogh C. M. P. M. (2012). Thoughts on the future: The perspectives of elderly people with early-stage Alzheimer's disease and the implications for advance care planning. AJOB Primary Research, 3(1), 14–22. DOI: 10.1080/21507716.2011.636784.

[bibr7-14713012241272852] Dening K. H. King M. Jones L. Sampson E. L. (2017). Healthcare decision-making: Past present and future, in light of a diagnosis of dementia. International Journal of Palliative Nursing, 23(1), 4–11. DOI: 10.12968/ijpn.2017.23.1.4. 28132606

[bibr8-14713012241272852] Eccleston C. Doherty K. Bindoff A. Robinson A. Vickers J. McInerney F. (2019). Building dementia knowledge globally through the Understanding Dementia Massive Open Online Course (MOOC). Npj Science of Learning, 4(1), 3–6. DOI: 10.1038/s41539-019-0042-4. 30993003 PMC6458180

[bibr9-14713012241272852] Edvardsson D. Winblad B. Sandman P. O. (2008). Person-centred care of people with severe Alzheimer's disease: Current status and ways forward. The Lancet Neurology, 7(4), 362–367. DOI: 10.1016/S1474-4422(08)70063-2. 18339351

[bibr27-14713012241272852] Goldman J. S. Trommer A. E. (2019). A qualitative study of the impact of a dementia experiential learning project on pre-medical students: A friend for Rachel. BMC Med Educ, 19(1), 127. DOI: 10.1186/s12909-019-1565-3. 31046761 PMC6498531

[bibr28-14713012241272852] Gove D. Downs M. Vernooij-Dassen M. Small N. (2016). Stigma and GPs’ perceptions of dementia. Aging & Mental Health, 20(4), 391–400. DOI: 10.1080/13607863.2015.1015962. 25765096

[bibr29-14713012241272852] Green G. Lakey L. (2013). Building dementia-friendly communities: A priority for everyone. Alzheimer’s Society. https://actonalz.org/sites/default/files/2023-01/Dementia_friendly_communities_full_report.pdf.

[bibr30-14713012241272852] Jeon Y.-H. Clemson L. Naismith S. L. Mowszowski L. McDonagh N. Mackenzie M. Dawes C. Krein L. Szanton S. L. (2018). Improving the social health of community-dwelling older people living with dementia through a reablement program. Int. Psychogeriatr, 30(6), 915–920. DOI: 10.1017/S1041610217001533. 28805186

[bibr31-14713012241272852] Jeon Y.-H. Simpson J. M. Low L. -F. Woods R. Norman R. Mowszowski L. Clemson L. Naismith S. L. Brodaty H. Hilmer S. Amberber A. M. Gitlin L. N. Szanton S. (2019). A pragmatic randomised controlled trial (RCT) and realist evaluation of the interdisciplinary home-based Reablement program (I-HARP) for improving functional independence of community dwelling older people with dementia: An effectiveness-implementation hybrid design. BMC Geriatr, 19(1), 199–114. DOI: 10.1186/s12877-019-1216-x. 31357949 PMC6664757

[bibr10-14713012241272852] Karel M. J. Moye J. Bank A. Azar A. R. (2007). Three methods of assessing values for advance care planning: Comparing persons with and without dementia. Journal of Aging and Health, 19(1), 123–151. DOI: 10.1177/0898264306296394. 17215205 PMC4859331

[bibr11-14713012241272852] Kitwood T. (1993). Person and process in dementia. International Journal of Geriatric Psychiatry, 8(7), 541–545. DOI: 10.1002/gps.930080702.

[bibr12-14713012241272852] Kitwood T. (1997). Dementia reconsidered the person comes first. Open University Press.

[bibr13-14713012241272852] Olson M. A. Kendrick R. V. (2012). Attitude Formation. In Ramachandran V. S. (Ed.), Encyclopedia of human behavior (2nd ed., pp. 230–235). Academic Press. DOI: 10.1016/B978-0-12-375000-6.00041-0.

[bibr14-14713012241272852] Peisah C. Bhatia S. Macnab J. Brodaty H. (2016). Knowledge translation regarding financial abuse and dementia for the banking sector: The development and testing of an education tool. International Journal of Geriatric Psychiatry, 31(7), 702–707. DOI: 10.1002/gps.4379. 26559928

[bibr15-14713012241272852] Phillipson L. Hall D. Cridland E. Fleming R. Brennan-Horley C. Guggisberg N. Frost D. Hasan H. (2019). Involvement of people with dementia in raising awareness and changing attitudes in a dementia friendly community pilot project. Dementia, 18(7-8), 2679–2694. DOI: 10.1177/1471301218754455. 29363336

[bibr32-14713012241272852] Poulos C. J. Bayer A. Beaupre L. Clare L. Poulos R. G. Wang R. H. Zuidema S. McGilton K. S. . (2017). A comprehensive approach to reablement in dementia. Alzheimers Dement (N Y), 3(3), 450–458. DOI: 10.1016/j.trci.2017.06.005. 29067351 PMC5654482

[bibr16-14713012241272852] Power G. A. (2014). Dementia beyond diseease: Enhancing wellbeing. Health Professions Press.

[bibr17-14713012241272852] Power G. A. (2017). Dementia beyond drugs: Changing the culture of care (2nd ed.). Health Professions Press, Inc.

[bibr18-14713012241272852] Prince M. Bryce R. Ferri C. (2011). The World Alzheimer Report 2011: The benefits of early diagnosis and intervention. https://www.alz.co.uk/research/WorldAlzheimerReport2011.pdf.

[bibr19-14713012241272852] Quinn C. Pickett J. A. Litherland R. Morris R. G. Martyr A. Clare L. On behalf of the IDEAL Programme Team . (2022). Living well with dementia: What is possible and how to promote it. International Journal of Geriatric Psychiatry, 37(1). DOI: 10.1002/gps.5627. PMC9292841 34564897

[bibr20-14713012241272852] Read S. Wynaden D. Albrecht M. A. Toye C. (2020). Development of the dementia community attitudes questionnaire. Dementia, 20(6), 1940–1957. DOI: 10.1177/1471301220977649. 33307762

[bibr21-14713012241272852] Read S. T. Toye C. Wynaden D. (2016). Experiences and expectations of living with dementia: A qualitative study. Collegian, 24(5), 427–432. DOI: 10.1016/j.colegn.2016.09.003.

[bibr22-14713012241272852] Surr C. A. Gates C. Irving D. Oyebode J. Smith S. J. Parveen S. Drury M. Dennison A. (2017). Effective dementia education and training for the health and social care workforce: A systematic review of the literature. Review of Educational Research, 87(5), 966–1002. https://www.jstor.org.ezproxy.ecu.edu.au/stable/44667682 28989194 10.3102/0034654317723305 PMC5613811

[bibr33-14713012241272852] Trainor A. . (2018). Community conversation as a method of gathering and analyzing qualitative data. Journal of Disability Policy Studies, 29, 104420731773940. DOI: 10.1177/1044207317739403.

[bibr23-14713012241272852] United Nations . (2006). Convention on the rights of persons with disabilities. https://www.un.org/disabilities/documents/convention/convoptprot-e.pdf. 10.1515/9783110208856.203 18348362

[bibr24-14713012241272852] Vernooij-Dassen M. Moniz-Cook E. Woods R. T. De Lepeleire J. Leuschner A. Zanetti O. de Rotrou J. Kenny G. Franco M. Peters V. Iliffe S. (2005). Factors affecting timely recognition and diagnosis of dementia across Europe: From awareness to stigma. International Journal of Geriatric Psychiatry, 20(4), 377–386. DOI: 10.1002/gps.1302. 15799080

[bibr25-14713012241272852] World Health Organisation . (2017). Global action plan on the public health response to dementia 2017–2025. https://apps.who.int/iris/bitstream/handle/10665/259615/9789241513487-eng.pdf?sequence=1

[bibr26-14713012241272852] World Health Organization . (2015). WHO global strategy on people-centred and integrated health services: Interim report. https://apps.who.int/iris/handle/10665/155002.

